# Psoriasis and chronic kidney disease: a systemic inflammatory link and a practical kidney assessment pathway

**DOI:** 10.1093/ckj/sfag254

**Published:** 2026-08-05

**Authors:** Gustavo Almeida-Silva, Marco Morais, Inês Tribolet-Abreu, Filipe Monteiro, João Ferreira, Joana Antunes, Paulo Filipe

**Affiliations:** Department of Dermatology, Hospital de Santa Maria, Unidade Local de Saúde Santa Maria, Lisbon, Portugal; Dermatology University Clinic, Faculty of Medicine, University of Lisbon, Lisbon, Portugal; Nephrology Department, Hospital de Santa Maria, Unidade Local de Saúde Santa Maria, Lisbon, Portugal; Department of Dermatology, Hospital de Santa Maria, Unidade Local de Saúde Santa Maria, Lisbon, Portugal; Dermatology University Clinic, Faculty of Medicine, University of Lisbon, Lisbon, Portugal; Department of Dermatology, Hospital de Santa Maria, Unidade Local de Saúde Santa Maria, Lisbon, Portugal; Dermatology University Clinic, Faculty of Medicine, University of Lisbon, Lisbon, Portugal; Dermatology University Clinic, Faculty of Medicine, University of Lisbon, Lisbon, Portugal; Department of Dermatology, Hospital de Santa Maria, Unidade Local de Saúde Santa Maria, Lisbon, Portugal; Dermatology University Clinic, Faculty of Medicine, University of Lisbon, Lisbon, Portugal; Department of Dermatology, Hospital de Santa Maria, Unidade Local de Saúde Santa Maria, Lisbon, Portugal; Dermatology University Clinic, Faculty of Medicine, University of Lisbon, Lisbon, Portugal

To the Editor,

Psoriasis is increasingly recognized as a systemic immune-mediated disease rather than a cutaneous disorder alone. Its pathophysiology is driven by persistent activation of the interleukin (IL)-23/IL-17 axis and tumour necrosis factor-alpha, with downstream inflammatory and cardiometabolic consequences that extend beyond the skin [1, 2].

This systemic perspective is clinically relevant for nephrologists because psoriasis frequently coexists with obesity, hypertension, diabetes, dyslipidaemia, and cardiovascular disease, all of which may contribute to kidney injury and chronic kidney disease (CKD) progression [3].

Accumulating epidemiological evidence supports an association between psoriasis and kidney disease, particularly in patients with moderate-to-severe disease. In a large population-based cohort, Wan *et al*. reported that moderate-to-severe psoriasis was associated with a higher risk of moderate-to-advanced CKD independent of traditional risk factors [4].

A nationwide Taiwanese cohort similarly found that severe psoriasis, but not mild psoriasis, was independently associated with incident CKD and kidney failure [5].

These data are supported by a systematic review and meta-analysis showing an increased risk of both incident CKD and kidney failure among patients with psoriasis [6].

The biological plausibility of this association is strong. Chronic systemic inflammation may promote endothelial dysfunction, oxidative stress, microvascular injury, and activation of pathways implicated in kidney fibrosis. In parallel, metabolic syndrome and related comorbidities, which are enriched in psoriasis, may act as mediators or amplifiers of kidney risk [2, 3, 7].

Treatment-related factors also matter: non-steroidal anti-inflammatory drugs (NSAIDs), cyclosporine and, in patients with reduced estimated glomerular filtration rate (eGFR) or significant albuminuria, methotrexate may accelerate harm or become unsafe, whereas systemic treatment decisions in established CKD require careful dose adjustment, avoidance of nephrotoxicity, and coordination between dermatology, rheumatology and nephrology.

Among conventional systemic agents, methotrexate deserves particular caution when eGFR is reduced. Because methotrexate is predominantly eliminated by the kidney, reduced eGFR, dehydration, intercurrent illness, hypoalbuminaemia, older age or frailty, and concomitant nephrotoxic or interacting drugs may convert a standard weekly dermatological or rheumatological dose into clinically dangerous exposure, with mucositis, myelosuppression, infection, pneumonitis, hepatotoxicity, and potentially fatal toxicity. Dermatology guidance identifies severe kidney dysfunction and dialysis as absolute contraindications to methotrexate, and a large population-based cohort of older adults with CKD found a higher 90-day risk of serious adverse events after low-dose methotrexate initiation, especially when eGFR was below 45 ml/min/1.73 m^2^ [9, 10].

NSAID overuse is also highly relevant in psoriatic arthritis, where repeated analgesic self-medication may coexist with inflammation, hypertension, diabetes, urate excess, and cardiovascular risk. NSAIDs inhibit prostaglandin-mediated afferent arteriolar vasodilation and may precipitate acute kidney injury, sodium and water retention, hyperkalaemia, and blood-pressure worsening, particularly in CKD or during dehydration and concomitant renin–angiotensin system inhibitor or diuretic use. In a prospective psoriatic arthritis cohort, daily NSAID use was independently associated with CKD development. For this pathway, when NSAID treatment is considered appropriate in CKD G1–G3, a brief course is defined as no more than five consecutive days; use beyond 5 days or recurrent daily or near-daily use over subsequent weeks is considered prolonged or recurrent exposure and should prompt clinician review and monitoring of eGFR, potassium, and blood pressure [11–13].

Despite this evidence, structured kidney screening is not consistently embedded in routine psoriasis care. Given the silent nature of early CKD, we propose a simple clinical pathway to identify kidney risk, stratify monitoring, and support safer systemic treatment selection in patients with psoriasis (see Figure 1).

**Figure 1:**
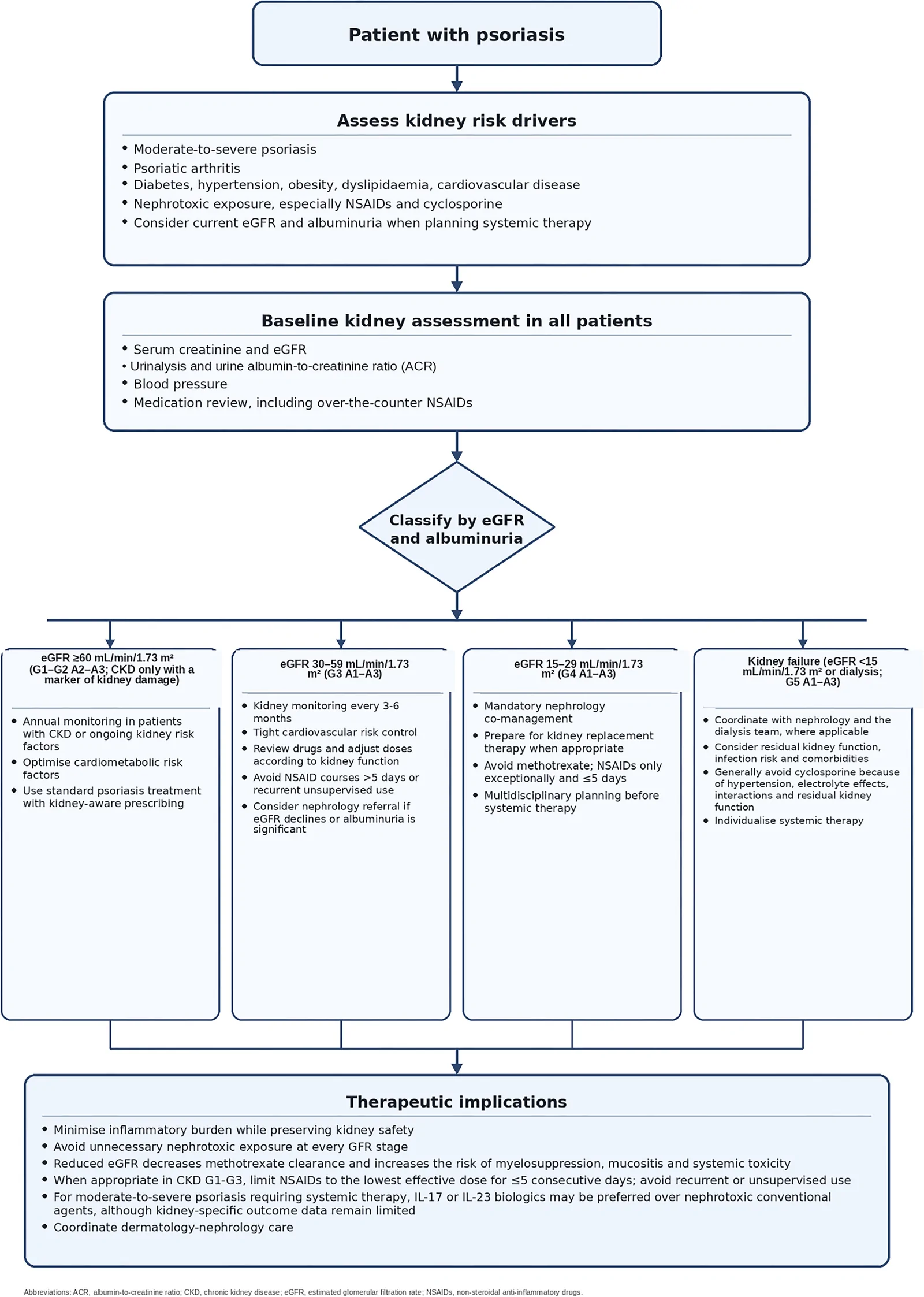
Proposed kidney risk assessment and integrated management pathway for psoriasis–CKD overlap. The algorithm begins with a diagnosis of psoriasis, identifies kidney risk drivers, recommends baseline kidney assessment, and stratifies management according to eGFR and albuminuria, including pathways for G1 and G2, CKD G3, CKD G4, and kidney failure. The model highlights avoidance of unnecessary nephrotoxic exposure at every GFR stage, coordinated dermatology–nephrology care, cardiometabolic risk optimization, explicit limits on NSAID exposure, and the preferential use of kidney-safer systemic therapies when clinically indicated.

## PROPOSED CLINICAL APPROACH TO KIDNEY RISK IN PATIENTS WITH PSORIASIS

**Initial evaluation—all patients with psoriasis**

- Assess psoriasis severity (mild, moderate, or severe).
- Evaluate comorbidities: diabetes, hypertension, obesity, dyslipidaemia ,and cardiovascular disease.
- Review exposure to nephrotoxic agents, particularly prescribed and over-the-counter NSAIDs, cyclosporine and methotrexate; document dose, frequency, duration, dehydration episodes, and concomitant renin–angiotensin system inhibitors or diuretics.
- Perform baseline kidney assessment: serum creatinine with eGFR, urinalysis with albumin-to-creatinine ratio when available, and blood-pressure measurement.

This approach aligns with contemporary CKD evaluation frameworks, which emphasize GFR assessment, albuminuria, and risk-based follow-up.

## CKD-BASED STRATIFICATION AND MANAGEMENT

**eGFR ≥60 ml/min/1.73 m^2^ (G1 and G2; CKD only if albuminuria or another marker of kidney damage is present)**

- Annual kidney monitoring in patients with CKD or ongoing kidney risk factors.
- Optimization of cardiometabolic risk factors.
- Use standard psoriasis treatment with attention to kidney function and albuminuria.

**eGFR 30–59 ml/min/1.73 m^2^ (CKD G3)**

- Kidney monitoring every 3–6 months, adjusted to albuminuria and rate of decline.
- Aggressive cardiovascular risk control.
- Medication review, including dose adjustment according to kidney function, avoidance of NSAID courses longer than five consecutive days or recurrent unsupervised use, and careful reconsideration or dose reduction of methotrexate according to eGFR and overall frailty.
- Consider nephrology referral if eGFR declines, albuminuria is significant or systemic psoriasis therapy is complex.

**eGFR 15–29 ml/min/1.73 m^2^ (CKD G4)**

- Mandatory nephrology co-management.
- Preparation for kidney replacement therapy when appropriate.
- Strict avoidance of avoidable nephrotoxins; methotrexate should generally be avoided, and NSAIDs should be used only exceptionally and for no more than five consecutive days, with analgesic and anti-inflammatory strategies planned jointly with nephrology/rheumatology.
- Multidisciplinary dermatology–nephrology planning before initiating or changing systemic psoriasis therapy.

**Kidney failure (eGFR <15 ml/min/1.73 m^2^ or dialysis)**

- Coordinate dermatology care with the nephrology and dialysis teams, where applicable.
- Prefer systemic agents with a favourable kidney safety profile when treatment is required.
- Generally, avoid cyclosporine because it may worsen hypertension and electrolyte disturbances, has a substantial drug-interaction burden, and can compromise clinically important residual kidney function; avoid methotrexate because of accumulation and severe toxicity risk.
- Monitor inflammatory burden, nutritional status, and pruritus severity.
- Individualize systemic therapy according to residual kidney function, kidney replacement therapy, infection risk, and comorbidity burden.

The systemic inflammatory burden of psoriasis should be minimized while preserving kidney safety. In patients with moderate-to-severe psoriasis who require systemic therapy, biologic agents, including IL-17 and IL-23 inhibitors, may be broadly preferred across CKD stages over directly nephrotoxic or substantially kidney-cleared conventional agents, provided that the usual treatment-specific contraindications are absent. These biologics are not regarded as a classically nephrotoxic drug class; monoclonal antibody clearance is not expected to depend substantially on glomerular filtration, and available observational studies have not shown deterioration of kidney function. However, dedicated trials in advanced CKD and dialysis remain limited, and there is no evidence that these drugs improve long-term kidney outcomes. Therefore, biologic selection should still account for psoriasis severity, psoriatic arthritis activity, inflammatory bowel disease, infection risk, prior treatment, access, and patient preference [14–16]. Methotrexate should not be viewed as a routine option when eGFR is reduced; if considered in mild-to-moderate CKD, it requires explicit dose adjustment, folate supplementation, avoidance of interacting drugs, close full blood count/liver/kidney monitoring, and patient education about stopping therapy during dehydration or acute illness. NSAID exposure should be avoided where possible at every GFR stage. When an NSAID is judged necessary in CKD G1–G3, use should be limited to the lowest effective dose for no more than five consecutive days; use in CKD G4 should be exceptional and closely supervised, and use in kidney failure generally avoided, with nephrology input [8, 13, 14].

Psoriasis should be considered a systemic inflammatory disorder associated with increased CKD risk, particularly in moderate-to-severe disease and in patients with psoriatic arthritis. We propose a pragmatic kidney assessment and CKD-stratified management pathway to improve early detection, reduce nephrotoxic exposure, identify hazardous methotrexate use in patients with reduced eGFR, limit NSAID overuse, and support safer systemic treatment selection. Prospective studies are needed to determine whether integrated dermatology–rheumatology–nephrology pathways and effective systemic control of psoriatic disease can improve long-term kidney outcomes.
